# Exploring the Impact of COVID-19 on Individuals with Eating Disorders in Saudi Arabia: A Thematic Analysis

**DOI:** 10.3390/bs13010069

**Published:** 2023-01-13

**Authors:** Aisha Jawed, Mengyu Lim, Amy Harrison, Gianluca Esposito, Nandini Adusumilli, Dagmara Dimitriou

**Affiliations:** 1 Sleep Education and Research Laboratory, UCL Institute of Education, London WC1H 0AA, UK; 2 Psychology Program, School of Social Sciences, Nanyang Technological University, 48 Nanyang Avenue, Singapore 639818, Singapore; 3 Department of Psychology and Human Development, University College London, 25 Woburn Square, London WC1H 0AA, UK; 4 Affiliative Behaviour and Physiology Lab, Department of Psychology and Cognitive Science, University of Trento, 38122 Trento, Italy

**Keywords:** anorexia nervosa, eating disorders, bulimia, binge eating disorder, COVID-19, sleep, thematic analysis

## Abstract

Food and sleep are critical for human survival. However, for individuals with eating disorders, they face two critical disruptions in terms of abnormal feeding patterns as well as poor sleep. These difficulties are exacerbated as a result of the recent coronavirus pandemic, which caused drastic changes in daily life schedules and living arrangements. The current study therefore aims to explore, through qualitative means, how individuals with eating disorders are affected during the subsequent lockdowns, with a particular emphasis on the pandemic’s impact on their sleep in Saudi Arabia. Two groups of participants were recruited: participants with eating disorders and healthy controls. Based on thematic analysis of focus group discussion data, it was found that both types of participants experienced poorer sleep and poorer mental health. Participants with eating disorders too showed a deterioration of their symptoms. However, healthy participants tended to show greater levels of recovery and coping. Based on the study findings, recommendations for future studies are made.

## 1. Introduction

As theorized by Maslow’s hierarchy of needs [1], all human beings must first satisfy primal, physiological needs such as food, clothing, and shelter. What is commonly left out from this level, however, is the need for humans to sleep. In fact, sleep has profound implications on daily functioning, especially on cognitive processes such as attention, memory, and executive function [2]. The biological needs for food and rest are deeply connected with each other, where dysregulation in one aspect may result in dysfunction in the other.

Eating disorders (ED) are a class of discrete mental disorders characterized by abnormal eating habits, with potential to cause major harm to the individual’s psychosocial functions and physical health [3]. EDs affect a significant proportion of the population, with up to 8.4% of women and 2.2% of men diagnosed with lifetime ED [4]. A similar review and meta-analysis by [5] stated that prevalence of ED in non-Western countries may also be underestimated. In fact, in the city of Riyadh, Saudi Arabia, up to 26.5% of young adults sampled show symptoms of ED [6]. EDs are associated with a variety of symptoms, but most worryingly, EDs are linked to elevated mortality rates and a lowered quality of life [7].

Anorexia nervosa (AN), bulimia nervosa (BN), and binge eating disorder (BED) are the three major and most well-defined types of eating disorders, according to the fifth edition of Diagnostic and Statistical Manual of Mental Disorders (DSM-5; [4]). In a recent review by Cooper and colleagues (2020) [8], the authors affirm the observation that individuals with EDs commonly report experience issues with sleep, which affects more than half of all patients diagnosed with ED [9]. It was reported that EDs are associated with several symptoms of poor sleep, such as insomnia, changes in duration of dreams and associated rapid eye movement, as well as decreased sleep efficiency [10]. Furthermore, in night eating syndrome, disturbances in sleep duration are directly related to abnormal eating behaviours, where the individual deliberately wakes up to eat [11]. However, reason for the common co-occurrence of sleep issues with EDs is not fully known. Specific studies into each ED and its associated sleep issues show conflicting results. For example, body mass index (BMI) was pointed as a potential cause for sleep problems among those with AN and BN [12], while older studies have also posited the potential effect of poor nutrition [13].

Nonetheless, it must be acknowledged that the exposure to both atypical feeding and sleep behaviours exact profound effects on individuals with ED. Sleep disturbances are a significant predictor of depressive and anxiety symptoms experienced by individuals with AN [14], indicating the heightened risk of co-morbidities. In fact, as observed by [15], individuals with ED have poorer sleep and worse quality of life even compared to those with mood disorders, which is a significant finding in and of itself due to the centrality of sleep impairment in defining mood disorders and not ED.

The recent coronavirus pandemic (COVID-19) had a big impact on the daily life cycles of individuals [16]. Taking into account the existing feeding and sleeping issues faced by individuals with ED, it can be expected that the impact of COVID-19 would be even greater. For example, Schlegl and colleagues (2020) [17] pointed out several features of the pandemic and resulting disease containment strategies that may be particularly damaging to individuals with ED, including limiting social contact only to social media, the closure of restaurants and stockpiling of food at home. In a subsequent study, Ref. [18] indeed found a worsening of symptoms and quality of life among patients with BN, a finding corroborated by studies conducted elsewhere in the world [19,20,21]. However, there is still a lack of data comparing experiences between ED and healthy populations during the pandemic, particularly in the domain of sleep.

Therefore, by using qualitative focus group discussion data, the present study aims to explore the impact of the COVID-19 pandemic and its lockdowns on individuals with ED, particularly in terms of their sleep, in comparison to healthy controls.

## 2. Materials and Methods

To address the research goal stated above, the present study implemented two rounds of qualitative data collection. The materials and protocol were approved by the University College London Institute of Education Research Ethics Committee (REC: 1227).

### 2.1. Participants

Twenty-four young adults (aged between 18 to 35 years; 6 male, 18 female) were recruited through convenience sampling from social media platforms (e.g., Facebook) and personal networks (i.e., by word of mouth and personal contacts such as friends, relatives, and members of the community) in Jeddah, Saudi Arabia. Jeddah is a port city in Saudi Arabia that is home to approximately 4 million residents. As an under-represented population of study within the field of eating disorders [22], the sample of participants from Saudi Arabia represented a precious source of data from the Middle East. All participants in this round of recruitment were clinically diagnosed with an ED (either AN, BN or BED) by a medical professional (e.g., medical doctor) prior to the pandemic. Participants were excluded if they fell out of the age range stated, or if they did not have an official ED diagnosis. Participants with other psychiatric comorbidities were not excluded from taking part in the study. Due to the small sample size, no differentiation was made between the nature of the ED in subsequent analyses. Data collection in this round took place between April and June 2020, in the initial wave of COVID-19. Participants were split into 4 different focus groups consisting of 5–9 individuals each. In the second round of data collection, eight young adults (aged between 18 to 35 years; 4 male, 4 female) without eating disorders was recruited to serve as healthy controls. Data collection in this round took place between June and July 2022, in the last waves of COVID-19. Participants were split into 2 different focus groups consisting of 4 individuals each.

### 2.2. Materials

A series of 12 focus group discussion questions were prepared and presented to all participants. Questions covered eating habits and sleep problems during the pandemic (and post-pandemic for the second wave of recruited participants), specifically in terms of (1) lifestyle changes, (2) relationship with body, (3) mental health, (4) daily functioning, (5) access to help and support, and (6) coping. The full list of questions may be made available upon request to the corresponding author.

### 2.3. Protocol

Both waves of participant recruitment adopted identical experimental protocols. Informed consent was obtained from all participants via email before inviting them to take part in the focus group discussion.

Due to the concerns of disease spread during COVID, all focus groups were conducted online via the Piazza platform (www.piazza.com, accessed on 1 April 2020), which is a question-and-answer forum presented in a bulletin board layout. Piazza has been previously used to facilitate academic discussions [23,24], as well as for previous qualitative analysis studies [25]. Participants were given an anonymous username and could log in and out of the forum as many times as they wished, at any time or location of their choosing. Participants were given a period of two weeks to take part in the focus group, during which they were allowed to create their own responses to the focus group discussion questions, or to reply to another participant’s answer. Questions can be answered in any order; indeed, it is not compulsory for participants to answer every question posed in the focus group. In the present study, Piazza was chosen as the mode of data collection for several reasons: (1) online modality to enable social distancing, (2) ease of access for participants living in different countries, (3) ability to freely contribute responses at participants’ convenience, and (4) anonymity. All focus group sessions were moderated by members of the research team.

### 2.4. Thematic Analysis

After the conclusion of each focus group session, all written responses posted on Piazza were transferred verbatim into an offline document. There, all data were analysed using qualitative thematic analysis, following the framework set out by [26]. First, data was read and re-read to ensure familiarity with its content, before line-by-line analysis. Then, data was sorted based on responses to each question posed and coded for relevancy. Responses which were repetitive were identified and categorised into initial thematic codes. Next, other similar or related responses were grouped into these codes using a thematic map, from which the final themes were identified, reviewed, and labelled. These themes were discussed and agreed upon by A.J., M.L., N.A., and D.D.

Findings are presented below in greater detail, together with supporting quotes, following the labels of the finalised themes. Where applicable, data from both ED and healthy populations were discussed in parallel.

## 3. Results and Discussion

**Figure 1:**
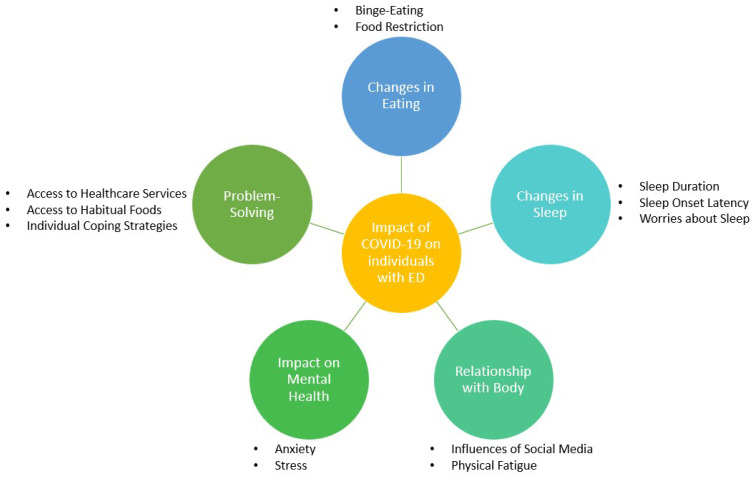
Thematic network of the impact of COVID-19 on individuals with eating disorders.

A total of five key themes were generated from thematic analysis, each with a varying number of further sub-themes to better illustrate the discrete issues reported by participants (Figure 1).

### 3.1. Theme 1: Changes in Eating

This theme was further sub-divided into changes in terms of binge-eating, as well as food restriction (e.g., eating less or experiencing decreased appetite). Expectedly, most participants with ED reported negative changes to their eating behaviour in corroboration with previous studies [18,20,27]. Some have taken measures to address or cope with these changes (further discussed in Theme 5), whilst only two participants with ED reported no/positive changes during the pandemic. For the individual who experienced positive changes in dietary habits, it was attributed to an increase in home cooked meals:

I got healthier and losing weight since all meals are home cooked. The lockdown has [taught] me how to cook better. (P7)

Although this theme is more relevant to participants with ED, it was also reported by healthy participants that changes in food intake and appetite were experienced. Therefore, where relevant, comparisons with healthy participants will be made.

#### 3.1.1. Binge-Eating

Some participants with ED reported increased episodes of binge-eating, particularly involving caffeine, mostly due to boredom and late-night snacking as a result of simultaneous changes in sleep behaviour (further discussed in Theme 2). The following quotes encapsulate emotional eating common in individuals with ED [28]:

However I drink coffee a lot, more than usual for sure. [Not] sure why, probably because I have nothing better to do. (P3)

I ate so much. Specially during the beginning as [I] had nothing else to do. An average of 7 meals a day. Also the fact that [I] was waking up at night, there was nothing else for me to do but cook and eat. (P11)

Recurrent episodes of binge-eating characteristic of BED were also experienced. As reported by P6:

At the beginning binge eating definitely, then controlled eating due to weight gain, and then back again to binge eating.

Three out of the eight healthy participants also experienced an increase in appetite and food consumed during the pandemic, with some pointing to emotional/boredom-related eating:

I am eating more than I used to as I find myself to be more anxious which leads to stress eating. Moreover, I seem to eat more out of boredom as I have more time in my hands now. (sic) (C7)

This result supports a meta-analysis indicating that the pandemic was a risk factor in the development of emotional eating [29], which may be attributed to increased distress during a challenging time [30]. Nonetheless, these increases in food intake seem only transient and participants have since recovered their typical eating habits post-pandemic:

My eating habits have increased drastically since the COVID-19 pandemic [started]… However, after the pandemic ordering food from outside [has] reduced. (C6)

#### 3.1.2. Food Restriction

Some participants with ED reported increased levels of food restriction in the form of reduced appetite and food intake. For example, P19 explained:

I used to eat normally though I do not eat much but now with COVID [I] am not able to eat much loss of appetite… and [I] am restricting and there is reduced food intake.

Some participants attributed this food restriction due to fears and anxiety about COVID-19:

I am basically restricting myself and trying to have less fear as I do not want to gain weight and the fear of COVID-19 is not letting me eat my appetite is so bad and [I] have so many issues. (P20)

I am not restricting but not able to eat properly though getting all the food I want and getting it prepared but not able to eat loss of hunger maybe… or is it toooo much thinking or what… [COVID] is affecting me. (P21)

Although responses from other participants did not explicitly mention food restriction, they reported difficulties with daily function, such as physical weakness, brain fog, skipping responsibilities, and low levels of concentration which may be indirect consequences of food restriction. Specifically, participants tended to brush off low energy levels as being “lazy” (see further discussion in Theme 3).

Healthy participants on the other hand reported mixed to positive responses. Most of the participants claimed to follow the same diet and in fact made use of the lockdown imposed during COVID-19 to make healthier choices. This improvement in diet was exemplified by C2:

[COVID-19] pandemic gave me a good time to change my unhealthy eating habits and lose some extra body weight which I wanted to for a while, but had been neglecting it. Making my own soups, salads, spreading out my meals and being more cautious about health and in general eating better were some things I did during the pandemic. Also, from the fear of not contracting the virus and having a better immune system ready. (sic)

There was only one healthy participant who mentioned loss of appetite, albeit only an acute episode in the beginning of the pandemic:

Initially, I lost my appetite where I would only have 2 meals a day, but now [I’ve] corrected my eating habits and I follow a proper nutritious diet. (C5)

### 3.2. Theme 2: Changes in Sleep

This theme was further sub-divided into changes in terms of sleep duration, trouble falling asleep (i.e., longer sleep onset latency), and worries regarding sleep problems. Generally, it was agreed by all that sleep quality had suffered during the pandemic, supporting a large meta-analysis that found up to 40% of the general population experiencing sleep issues during COVID-19 [31].

#### 3.2.1. Sleep Duration

Participants with ED reported poorer sleeping schedules during the pandemic. However, while all report negative changes in sleep duration, the direction of change is inconsistent. For example, some participants mentioned increased sleep duration:

At first, I was sleep deprived no sleep at all (sic), then it turned into too much sleep. (E2)

On the other hand, others mentioned a decrease in the number of hours spent asleep due to changes in work arrangements:

First two months I was not sleeping properly (4–5 [hours] daily max and no naps) because I had to wake up super early to do work from home. (E1)

These changes were similarly found among healthy participants who reported altered and more irregular sleep schedules, including sleeping for longer and taking naps during the day:

I find it hard to maintain a regular sleep schedule and struggle with going to bed and waking up at consistent times. (C4)

This pattern of altered sleep schedules and increased napping was also found in [32], whose data collection was conducted in approximately the same period of the pandemic.

#### 3.2.2. Sleep Onset Latency

Participants with ED reported trouble falling asleep, citing high levels of “insomnia”, “physical exhaustion” and experiencing negative thoughts before sleep (elaborated in the next sub-theme). Not all healthy participants, however, reported trouble falling asleep. Nonetheless, among those who did, participants appeared to have classic symptoms of acute insomnia, citing “trouble in both falling asleep as well as staying asleep” (C1).

#### 3.2.3. Worries about Sleep

Some participants with ED highlighted feeling heightened worry during the pandemic that included uncertainty about the future, bad dreams, and fear of their ED relapsing, that affected their sleep quality. P8 listed these worries succinctly:

My main problem in getting to sleep is: overthinking, stressing about work, calculating how many hours I need to sleep and worry that it is not enough, afraid of oversleeping. (sic)

Another distinct worry was due to the lack of knowledge of why participants with ED were experiencing changes in their sleep behaviours, as well as how best to address these problems during a lockdown. For example, P21 said “I would like to know the hours of sleep that my body needs and how I can organize my body to sleep at a specific time every day. I would also like to know if having a organized eating schedule may amount to better sleep”.

Healthy participants did not report similar worries regarding knowledge gaps despite also experiencing worries about sleep. For example, despite having “numerous thoughts regarding different aspects of life occupy [their] mind” before sleep, C2 reports no extraneous concerns regarding their causes or solutions.

### 3.3. Theme 3: Relationship with Body

This theme was further sub-divided into influences of social media and physical fatigue.

#### 3.3.1. Influences of Social Media

Participants with ED focused on weight changes and changing perceptions of one’s body over the course of the pandemic. They reported instances of weight fluctuations as well as societal and internal pressures on body transformations and ’working out’ during the pandemic. As the only source of social interaction beyond the home, participants were particularly affected by content posted on social media platforms. As described by P14:

Yes, I used to feel bad whenever I see people exercising on social media. People kept posting home workouts and I couldn’t get myself to work out after all the gyms closed.

For some, these social pressures led to increased self-loathing and exacerbated the lack of motivation to maintain an active lifestyle due to lockdown. For example, P19 cites “stress stress stress and am losing weight and look ugly”, while P22 reports “my shape is [the] same or I do not know. [I’m] not seeing myself in the mirror [because I] do not want to freak out - and yes the fact that [I] cannot go to gyms is affecting me mentally. [I] try to work out at home but [it’s] not the same feeling”. It is already known that social media plays a large part in shaping and predicting ED behaviours [33,34] due to their tendency to promote social comparison [35], particularly content that focused on “fitspiration” [36]. Within the context of COVID-19, it was found that exposure to fitness-related content on social media led to greater appearance anxiety during this period [37], thereby corroborating the present study findings.

#### 3.3.2. Physical Fatigue

In addition to social media-driven changes in body perception, most participants with ED reported a general sense of fatigue, exemplified by responses from P6 and P21:

Fatigue definitely. I feel the need to have a massage constantly. The laziness makes the body feel so tired. (P6)

I am so lazy if I sit in one place then [I] am stuck there… have no motivation to move around, [I] do feel tired. (P21)

In addition to a lack of energy, the labelling of these emotions as a state of “laziness” is characteristic of self-criticism that is common among individuals with ED [38]. Nonetheless, these problems related to a lack of energy and motivation appeared to get worse as the pandemic progressed:

In the beginning [I] was over-active [I] had [a lot] of energy that [I didn’t] know what to do with so [I] would find different things to do. Then my body started to become lazy and all [I] wanted to do was relax. (P7)

For healthy participants, similar decreases in physical energy and fatigue were also experienced. As C2 explains, they “kind of feel more lazy, fatigued and not as energized to get the day going, as [they] tend to be indoors with the same set of individuals in [their] comfort zone”. However, despite a general sense of tiredness, 5 of 8 healthy participants cited no change in the relationship with their bodies, while only 1 mentioned a feeling of being “less fit” (C8). The other 2 healthy participants in fact mentioned a positive development in body image, with C4 claiming that they were in “better shape that [they] had ever been before”.

### 3.4. Theme 4: Impact on Mental Health

This theme was further sub-divided into mental health impact in terms of anxiety, as well as stress levels. Generally, while both categories of participants agreed that their mental health was negatively affected because of the pandemic, participants with ED reported a worsening of their mental health status over time, while healthy participants reflected a psychological adaptation to the ’new normal’, with corresponding improvements in mental health over time. In comparison to the findings reported in [20], where both ED and healthy groups showed a worsening of depressive symptoms over time, the data captured in our healthy population showed a capacity for recovery post-COVID-19, perhaps because their causes of poor mental health were acute but transient.

#### 3.4.1. Anxiety

Participants with ED reported an increase in anxiety as the pandemic progressed, pointing to changes in “routine, [nutrition] and [not being able to exercise]” (P21). Some participants mentioned an increase in panic attacks (P26, P27). P21 even went so far as to describe their state of psychological health as “maybe I’m going mental”. Other participants in this category mentioned a worsening of co-morbid obsessive-compulsive disorder symptoms and meta-worrying characteristic of generalised anxiety disorders:

My OCD became worse at first and [I] didn’t want to touch anything. (P10)

I have become more anxious and stressed (which is a lot considering I’m already constantly worried). (P14)

Too much stress and I have a worry what if I get Chronic stress so worried do not know what to do. (sic) (P20)

Conversely, although healthy participants also experienced increases in anxiety due to the pandemic (e.g., the uncertainty of “not being able to go out”; C4), most of their concerns stemmed not from caloric intake and expenditure but from academic- or work-related changes. As put by one of the participants:

Stress and anxiety don’t play a major role… My mood solely depends on the work I have the next day. (C6)

#### 3.4.2. Stress

Participants with ED reported spikes in stress levels that are accompanied by decreased motivation and mood swings, although negative mood dominated their emotional states:

Mood was better in the beginning, but the longer this lasts the longer I don’t know when it will finish… making me more anxious with time. (P2)

At the start of the pandemic I was fine… but as time passed by I became less motivated and very anxious. (P4)

Since the start of the pandemic my mood has been temperamental, sometimes I feel fine and happy, other times I have anxiety and feel terrible. (P23)

Even though participants with ED had made attempts to implement coping strategies (further discussed in Theme 5), “some stress is [still] involved” (P6). On the other hand, while healthy participants mentioned similar struggles with stress, they seemed to have an easier time adapting to the changes in lifestyle routine, and with it an improvement in mental health:

As we got accustomed to the new normal, I saw my mood, mental health and sleep all get better with time. (C4)

### 3.5. Theme 5: Problem-Solving

This theme was further sub-divided into access to helpful resources such as healthcare services and habitual foods, as well as coping strategies implemented by participants to help themselves through the difficult situation of the pandemic.

#### 3.5.1. Access to Healthcare Services

In terms of online or offsite therapeutic services, it should be noted that although they have potential to benefit individuals with ED, Schlegl and colleagues (2020) [17] recommended a tailoring of these services to better account for the increased depressive and anxiety symptoms experienced by clients during COVID-19, as well as on tolerating uncertainty and emotional regulation [39]. In our study, participants with ED reported mixed levels of access to doctors and medical treatment. While some participants reported having had consistent access, the majority expressed decreased availability of doctors and ability to make appointments. Their responses included problems of COVID-flooded hospitals (P19, P20, P21) and unhelpful doctors (P7, P9), best explained by P7’s comment:

I do have access to my doctors online. However, it’s not really helpful to have them online. It is good that I can contact them whenever I have a concern, nevertheless it always ends up by needing to physically go for a visit.

These sentiments were echoed by healthy participants, who mostly reported having access to only online access to doctors (C1-7). However, a majority of healthy participants have not needed to consult medical professionals during lockdown and thus had no personal experiences with navigating these online services.

#### 3.5.2. Access to Habitual Foods

Responses in this category diverged depending on participants’ usual eating arrangements. Participants who were more reliant on home-cooked meals were less affected (P7, P8). Participants who used to order food frequently were the hardest hit, reporting decreased access to their regular food choices and altered eating arrangements as a result of the lockdown. In addition to sanitation concerns where participants were now more wary of their sources of food, the lockdown also imposed practical barriers to being able to obtain take-out and sometimes delivery. P12 recounts:

This was one of worst effects the pandemic had on me. I usually like to order in at least once a day, but during the pandemic I wasn’t able, not only because I had reservations regarding the sanitation and so on but mainly also due to my sleeping- pattern I always missed the curfew and wasn’t able to order in. I would get up so late that I witnessed the beginning of the curfew. (sic)

This led to attempts by some participants to pivot to home-cooked food (discussed further in the next sub-theme).

For healthy participants, although some participants described the initial difficulty in accessing habitual food options, most reported no issues. In fact, with C6, the lockdown was an opportunity to discover new food choices:

With the comfort of ordering food online during the start of COVID-19, I discovered new cloud kitchens which made me discover new food items and made it much more accessible.

#### 3.5.3. Individual Coping Strategies

To tackle the various difficulties and changes in sleeping and eating behaviours illustrated above, participants with ED reported many ways in which they attempted to manage their condition.

In coping with changes with eating habits and alterations with eating and delivery options, participants with ED have attempted to make healthier choices with food. This strategy was exemplified by P22:

I become more aware of what food I should consume on the daily basis, I started eating healthier and taking lots of vitamins to boost my immune system. (sic)

In terms of coping with sleep changes, participants mostly tried the forceful ’resetting’ of sleep cycles by avoiding naps, changing sleep timings, and pulling multiple all-nighters. As described by P18:

At first my sleeping habits were very bad, I don’t know my day from night and this feeling made me very depressed so I tried to push myself to sleep at 12 am and wake up around 10 am… it took a week for me to settle but in the end it was the best thing I have done in quarantine. (sic)

Additionally, the creation of fixed routines or to-do lists for themselves appeared to help with regulating sleep hours. This aligns with previously reported helpful coping behaviour in Schlegl and colleagues (2020) [17]. As reported by P13:

I figured a way to get through it by scheduling my day. I woke up at 10:00 am do my work out, eat breakfast, read a book, eat lunch in a specific time after that my sleeping and eating habits changed and the day goes faster than expected. (sic)

However, attempts to self-medicate were less successful, which may also lead to a deterioration of ED symptomatology due to poor coping [40]:

At times I try to take Panadol Night or even Red Bull so I can be awake and sleep for long the next day but it does not help at all (sic). (P20)

To improve their daily affect and mental health, participants reported that prioritising the family and re-evaluating their values were somewhat helpful:

I’m always trying to look at the positive side of this pandemic by spending more time with my family. Finish tasks that I always wanted to achieve by reading more books organize the house and see what is missing. Rethinking priorities and always be thankful of the small things you have. At last the pandemic is something stressful and I’m still having mood swings, but I’m getting very comfortable staying at home. (sic) (P7)

In fact, this sentiment was echoed by Cooper and colleagues (2022) [41] who recommended more enjoyable or valued activities during COVID-19.

Healthy participants also mentioned many coping techniques they have developed for themselves that included meditation before sleep (C1, C5), aroma therapy and essential oils at bedtime (C2) as well as avoiding over eating before sleep (C6, C8).

### 3.6. Study Evaluation and Future Recommendations

This qualitative study explored the impact of the COVID-19 pandemic and its lockdowns on individuals with ED, particularly in terms of their sleep, in comparison to healthy controls. Data from this study represented a unique period in modern human history, being a shared experience of public health crisis and social isolation across the world. Additionally, data was collected from the typically under-represented region of the Middle East, representing an attempt to fill in a significant research gap in understanding individuals from non-WEIRD nations [42].

However, the study is not without its limitations. Firstly, due to effects of social desirability bias and social dynamics during a focus group discussion, these self-reported responses may show bias. Although these biases may be mitigated by the fact of guaranteed anonymity over online platforms, it appears that these effects can still be detected [43]. To overcome this problem, Koivula and colleagues (2019) [44] recommend a multi-modal approach to data collection, which may be considered for future studies. Building on the multi-modal approach to data collection, future research may wish to adopt both qualitative and quantitative methods to document sleep patterns. For example, the use of actigraphy and portable electroencephalography can be used by participants to record objective, real-time sleep behaviours. Other subjective measures such as food and sleep diaries can also be implemented to complement data from qualitative discussions.

Finally, due to the already small sample size, the participants were not further segmented into the types of ED they were diagnosed with. Additionally, other than the self-disclosure of OCD symptoms by one of the participants, it was unclear if other participants with ED had co-morbid disorders. Given that co-morbidities (e.g., anxiety disorders) may also be a significant contributor to problems faced in sleep and eating patterns [45,46], it is recommended that future studies also take secondary medical or psychiatric conditions into consideration.

Overall, findings from this study revealed the added vulnerabilities of individuals with ED, particularly due to the unique characteristics of the pandemic and its accompanying lockdowns that exacerbate this population’s existing sleep and feeding problems. Furthermore, the rise of social media as one of the few means of social interaction available during the pandemic meant that the negative effects of online social comparison are more apparent than ever. In light of these study findings, clinicians need to consider appropriate adaptations of traditional psychotherapies into an online format, while taking into account the unique characteristics of the pandemic in shaping these individuals’ mood and motivation. Additionally, they may also consider addressing the effects of social media during treatment.

## 4. Conclusions

The present study set out to explore the impact of COVID-19 on the lives of individuals with ED. Particularly, we focused on individuals’ experiences with lifestyle changes, mental health, perceptions of helpful resources available and their coping strategies. Two waves of data collection were conducted, recruiting both participants with ED and healthy controls. Results showed that both groups of participants experienced poor sleep and disruption of daily schedules as a result of the pandemic. This was also accompanied by poorer mental health. However, where healthy participants tended to show more resilience and greater recovery trajectories, the same remains to be seen among participants with ED. While both groups implemented coping strategies according to their means during COVID-19-related lockdowns, some improvements to individuals’ access to healthcare can be made.

## Data Availability

Full transcripts of data obtained during the focus group discussions may be made available upon request to the corresponding author.

## Abbreviations

The following abbreviations are used in this manuscript:

ED	Eating Disorders
AN	Anorexia Nervosa
BN	Bulimia Nervosa
BED	Binge-eating Disorder
DSM-5	fifth edition of Diagnostic and Statistical Manual of Mental Disorders
BMI	Body Mass Index

## References

[1] Maslow A.H. (1958). A Dynamic Theory of Human Motivation. Understanding Human Motivation.

[2] Worley S.L. (2018). The extraordinary importance of sleep: The detrimental effects of inadequate sleep on health and public safety drive an explosion of sleep research. Pharm. Ther..

[3] Schmidt U., Adan R., Böhm I., Campbell I.C., Dingemans A., Ehrlich S., Elzakkers I., Favaro A., Giel K., Harrison A. (2016). Eating disorders: The big issue. Lancet Psychiatry.

[4] Galmiche M., Déchelotte P., Lambert G., Tavolacci M.P. (2019). Prevalence of eating disorders over the 2000–2018 period: A systematic literature review. Am. J. Clin. Nutr..

[5] Qian J., Wu Y., Liu F., Zhu Y., Jin H., Zhang H., Wan Y., Li C., Yu D. (2022). An update on the prevalence of eating disorders in the general population: A systematic review and meta-analysis. Eat. Weight Disord.-Stud. Anorexia Bulim. Obes..

[6] Sami M.H., Alhafi M.M., Alshehri F.T., Alghamdi M.M., Aldawsari M.S., Rokon A.E., Alsumairi S.A., Alkhalefa B.K., Masuadi E. (2022). Prevalence of the Alarming Symptoms of Eating Disorders Among KSAU-HS Students, Riyadh, Saudi Arabia. Middle East J. Fam. Med..

[7] Winkler L.A.D., Christiansen E., Lichtenstein M.B., Hansen N.B., Bilenberg N., Støving R.K. (2014). Quality of life in eating disorders: A meta-analysis. Psychiatry Res..

[8] Cooper A.R., Loeb K.L., McGlinchey E.L. (2020). Sleep and eating disorders: Current research and future directions. Curr. Opin. Psychol..

[9] Kim K.R., Jung Y.C., Shin M.Y., Namkoong K., Kim J.K., Lee J.H. (2010). Sleep disturbance in women with eating disorder: Prevalence and clinical characteristics. Psychiatry Res..

[10] Lundgren J.D., O’Reardon J.P., Allison K.C., Spresser C.D. (2008). Sleep and quality of life in eating disorders. Sleep and Quality of Life in Clinical Medicine.

[11] Allison K.C., Spaeth A., Hopkins C.M. (2016). Sleep and eating disorders. Curr. Psychiatry Rep..

[12] Abdou T.A., Esawy H.I., Mohamed G.A.R., Ahmed H.H., Elhabiby M.M., Khalil S.A., El-Hawary Y.A. (2018). Sleep profile in anorexia and bulimia nervosa female patients. Sleep Med..

[13] Delvenne V., Goldman S., De Maertelaer V., Simon Y., Luxen A., Lotstra F. (1996). Brain hypometabolism of glucose in anorexia nervosa: Normalization after weight gain. Biol. Psychiatry.

[14] Rief W., Fichter M. (1992). The Symptom Check List SCL-90-Rand and Its Ability to Discriminate between Dysthymia, Anxiety Disorders, and Anorexia Nervosa. Psychopathology.

[15] De la Rie S., Noordenbos G., Van Furth E. (2005). Quality of life and eating disorders. Qual. Life Res..

[16] Casagrande M., Favieri F., Tambelli R., Forte G. (2020). The enemy who sealed the world: Effects quarantine due to the COVID-19 on sleep quality, anxiety, and psychological distress in the Italian population. Sleep Med..

[17] Schlegl S., Maier J., Meule A., Voderholzer U. (2020). Eating disorders in times of the COVID-19 pandemic—Results from an online survey of patients with anorexia nervosa. Int. J. Eat. Disord..

[18] Schlegl S., Meule A., Favreau M., Voderholzer U. (2020). Bulimia nervosa in times of the COVID-19 pandemic—Results from an online survey of former inpatients. Eur. Eat. Disord. Rev..

[19] McCombie C., Austin A., Dalton B., Lawrence V., Schmidt U. (2020). “Now It’s just old habits and misery”—Understanding the impact of the COVID-19 pandemic on people with current or life-time eating disorders: A qualitative study. Front. Psychiatry.

[20] Phillipou A., Meyer D., Neill E., Tan E.J., Toh W.L., Van Rheenen T.E., Rossell S.L. (2020). Eating and exercise behaviors in eating disorders and the general population during the COVID-19 pandemic in Australia: Initial results from the COLLATE project. Int. J. Eat. Disord..

[21] Termorshuizen J.D., Watson H.J., Thornton L.M., Borg S., Flatt R.E., MacDermod C.M., Harper L.E., van Furth E.F., Peat C.M., Bulik C.M. (2020). Early impact of COVID-19 on individuals with self-reported eating disorders: A survey of 1,000 individuals in the United States and the Netherlands. Int. J. Eat. Disord..

[22] Jawed A., Harrison A., Dimitriou D. (2020). The presentation of eating disorders in Saudi Arabia. Front. Psychol..

[23] Kolluru S., Varughese J.T. (2017). Structured academic discussions through an online education-specific platform to improve Pharm. D. students learning outcomes. Curr. Pharm. Teach. Learn..

[24] Carroll J.M., Wu Y., Shih P.C., Zheng S. (2016). Re-appropriating a question/answer system to support dialectical constructivist learning activity. Educ. Technol. Res. Dev..

[25] Sarrett J.C. (2018). Autism and accommodations in higher education: Insights from the autism community. J. Autism Dev. Disord..

[26] Braun V., Clarke V. (2012). Thematic Analysis.

[27] Fernández-Aranda F., Casas M., Claes L., Bryan D.C., Favaro A., Granero R., Gudiol C., Jiménez-Murcia S., Karwautz A., Le Grange D. (2020). COVID-19 and implications for eating disorders. Eur. Eat. Disord. Rev..

[28] Braden A., Musher-Eizenman D., Watford T., Emley E. (2018). Eating when depressed, anxious, bored, or happy: Are emotional eating types associated with unique psychological and physical health correlates?. Appetite.

[29] Burnatowska E., Surma S., Olszanecka-Glinianowicz M. (2022). Relationship between mental health and emotional eating during the COVID-19 pandemic: A systematic review. Nutrients.

[30] Cecchetto C., Aiello M., Gentili C., Ionta S., Osimo S.A. (2021). Increased emotional eating during COVID-19 associated with lockdown, psychological and social distress. Appetite.

[31] Jahrami H., BaHammam A.S., Bragazzi N.L., Saif Z., Faris M., Vitiello M.V. (2021). Sleep problems during the COVID-19 pandemic by population: A systematic review and meta-analysis. J. Clin. Sleep Med..

[32] Gupta R., Grover S., Basu A., Krishnan V., Tripathi A., Subramanyam A., Nischal A., Hussain A., Mehra A., Ambekar A. (2020). Changes in sleep pattern and sleep quality during COVID-19 lockdown. Indian J. Psychiatry.

[33] Padín P.F., González-Rodríguez R., Verde-Diego C., Vázquez-Pérez R. (2021). Social media and eating disorder psychopathology: A systematic review. Cyberpsychol. J. Psychosoc. Res. Cyberspace.

[34] Cataldo I., Lepri B., Neoh M.J.Y., Esposito G. (2021). Social media usage and development of psychiatric disorders in childhood and adolescence: A review. Front. Psychiatry.

[35] Ferguson C.J., Muñoz M.E., Garza A., Galindo M. (2014). Concurrent and prospective analyses of peer, television and social media influences on body dissatisfaction, eating disorder symptoms and life satisfaction in adolescent girls. J. Youth Adolesc..

[36] Cataldo I., De Luca I., Giorgetti V., Cicconcelli D., Bersani F.S., Imperatori C., Abdi S., Negri A., Esposito G., Corazza O. (2021). Fitspiration on social media: Body-image and other psychopathological risks among young adults. A narrative review. Emerg. Trends Drugs, Addict. Health.

[37] Cataldo I., Burkauskas J., Dores A.R., Carvalho I.P., Simonato P., De Luca I., Gómez-Martínez M.Á., Ventola A.R.M., Demetrovics Z., Szabo A. (2022). An international cross-sectional investigation on social media, fitspiration content exposure, and related risks during the COVID-19 self-isolation period. J. Psychiatr. Res..

[38] Thew G.R., Gregory J.D., Roberts K., Rimes K.A. (2017). The phenomenology of self-critical thinking in people with depression, eating disorders, and in healthy individuals. Psychol. Psychother. Theory, Res. Pract..

[39] Vuillier L., May L., Greville-Harris M., Surman R., Moseley R.L. (2021). The impact of the COVID-19 pandemic on individuals with eating disorders: The role of emotion regulation and exploration of online treatment experiences. J. Eat. Disord..

[40] Baenas I., Caravaca-Sanz E., Granero R., Sánchez I., Riesco N., Testa G., Vintró-Alcaraz C., Treasure J., Jiménez-Murcia S., Fernández-Aranda F. (2020). COVID-19 and eating disorders during confinement: Analysis of factors associated with resilience and aggravation of symptoms. Eur. Eat. Disord. Rev..

[41] Cooper M., Reilly E.E., Siegel J.A., Coniglio K., Sadeh-Sharvit S., Pisetsky E.M., Anderson L.M. (2022). Eating disorders during the COVID-19 pandemic and quarantine: An overview of risks and recommendations for treatment and early intervention. Eat. Disord..

[42] Henrich J., Heine S.J., Norenzayan A. (2010). The weirdest people in the world?. Behav. Brain Sci..

[43] Dodou D., de Winter J.C. (2014). Social desirability is the same in offline, online, and paper surveys: A meta-analysis. Comput. Hum. Behav..

[44] Koivula A., Räsänen P., Sarpila O. (2019). Examining social desirability bias in online and offline surveys. International Conference on Human-Computer Interaction.

[45] Levinson C.A., Rodebaugh T.L. (2012). Social anxiety and eating disorder comorbidity: The role of negative social evaluation fears. Eat. Behav..

[46] Ramsawh H.J., Stein M.B., Belik S.L., Jacobi F., Sareen J. (2009). Relationship of anxiety disorders, sleep quality, and functional impairment in a community sample. J. Psychiatr. Res..

